# Supplementary material for: LSD600: the first corpus of biomedical abstracts annotated with lifestyle–disease relations
Source: Database (Oxford). 2025 Jan 17;2025:baae129. doi: 10.1093/database/baae129 (PMC11756709; doi:10.1093/database/baae129)
Supplement: baae129_Supp [file baae129_supp.zip › suppl_data/CleanSupplementaryTable6.docx]

Supplementary Table 6. Statistics of label confusion by the model for all predicted relations in the test set.

| **Predictions** | Negative | Positive SA* | SA* | No SA | Treats | Negative SA* | Causes | Prevents | Controls |
| --- | --- | --- | --- | --- | --- | --- | --- | --- | --- |
| **Annotations** |  |  |  |  |  |  |  |  |  |
| Controls | 2 | 0 | 0 | 0 | 2 | 0 | 0 | 1 | 2 |
| Causes | 38 | 4 | 0 | 0 | 0 | 0 | 30 | 0 | 0 |
| Prevents | 6 | 0 | 0 | 0 | 0 | 7 | 0 | 10 | 0 |
| Treats | 20 | 0 | 0 | 0 | 21 | 1 | 0 | 0 | 1 |
| SA | 33 | 2 | 38 | 0 | 0 | 0 | 4 | 0 | 0 |
| Positive SA* | 16 | 71 | 2 | 0 | 0 | 0 | 2 | 1 | 0 |
| Negative SA* | 6 | 1 | 0 | 0 | 0 | 31 | 0 | 0 | 0 |
| No SA* | 4 | 0 | 0 | 21 | 0 | 0 | 0 | 0 | 0 |
| * SA: Statistical Association | | | | | | | | | |
