# Supplementary material for: LSD600: the first corpus of biomedical abstracts annotated with lifestyle–disease relations
Source: Database (Oxford). 2025 Jan 17;2025:baae129. doi: 10.1093/database/baae129 (PMC11756709; doi:10.1093/database/baae129)
Supplement: baae129_Supp [file baae129_supp.zip › suppl_data/SupplementaryTable1.docx]

Supplementary Table 1: Corpus statistics.

| Relation | Total | Train | Dev | Test |
| --- | --- | --- | --- | --- |
| *Statistical Association* | 287 | 148 | 62 | 77 |
| *Positive Statistical Association* | 592 | 419 | 81 | 92 |
| *Causes* | 295 | 128 | 95 | 72 |
| *Negative Statistical Association* | 279 | 177 | 64 | 38 |
| *Controls* | 60 | 31 | 23 | 6 |
| *Prevents* | 124 | 55 | 46 | 23 |
| *Treats* | 115 | 46 | 26 | 43 |
| *No Statistical Association* | 148 | 95 | 28 | 25 |
| **Total** | **1900** | **1099** | **425** | **376** |
