# Supplementary material for: LSD600: the first corpus of biomedical abstracts annotated with lifestyle–disease relations
Source: Database (Oxford). 2025 Jan 17;2025:baae129. doi: 10.1093/database/baae129 (PMC11756709; doi:10.1093/database/baae129)
Supplement: baae129_Supp [file baae129_supp.zip › suppl_data/SupplementaryTable2.docx]

Supplementary Table 2. Distribution of relations in train, development and test sets.

| number of relations | number of train set abstracts | number of dev set abstracts | number of test set abstracts |
| --- | --- | --- | --- |
| 0 | 162 | 56 | 59 |
| 1 | 22 | 8 | 8 |
| 2 | 38 | 5 | 5 |
| 3 | 26 | 5 | 7 |
| 4 | 16 | 4 | 8 |
| 5 | 18 | 10 | 8 |
| 6 | 21 | 7 | 8 |
| 7 | 13 | 5 | 6 |
| 8 | 12 | 6 | 2 |
| 9 | 8 | 3 | 1 |
| 10 | 4 | 2 | 0 |
| 11 | 5 | 1 | 1 |
| 12 | 2 | 1 | 0 |
| 13 | 2 | 1 | 1 |
| 14 | 1 | 2 | 0 |
| 15 | 2 | 0 | 1 |
| 16 | 1 | 0 | 0 |
| 17 | 0 | 1 | 0 |
| 18 | 2 | 1 | 0 |
| 20 | 0 | 0 | 2 |
| 21 | 0 | 0 | 1 |
| 22 | 1 | 1 | 0 |
| 23 | 1 | 0 | 0 |
| 24 | 0 | 0 | 1 |
| 26 | 1 | 0 | 1 |
| 33 | 0 | 1 | 0 |
| 36 | 2 | 0 | 0 |
