# Supplementary material for: LSD600: the first corpus of biomedical abstracts annotated with lifestyle–disease relations
Source: Database (Oxford). 2025 Jan 17;2025:baae129. doi: 10.1093/database/baae129 (PMC11756709; doi:10.1093/database/baae129)
Supplement: baae129_Supp [file baae129_supp.zip › suppl_data/SupplementaryTable3.docx]

Supplementary Table 3. Distribution of Lifestyle Factor mentions in train, development and test sets.

| number of LSF mentions | number of train set abstracts | number of dev set abstracts | number of test set abstracts |
| --- | --- | --- | --- |
| 0 | 17 | 6 | 6 |
| 1 | 13 | 2 | 6 |
| 2 | 11 | 3 | 5 |
| 3 | 19 | 2 | 6 |
| 4 | 8 | 4 | 4 |
| 5 | 17 | 5 | 9 |
| 6 | 10 | 8 | 2 |
| 7 | 23 | 9 | 7 |
| 8 | 21 | 4 | 7 |
| 9 | 25 | 6 | 7 |
| 10 | 19 | 5 | 6 |
| 11 | 14 | 8 | 5 |
| 12 | 23 | 5 | 4 |
| 13 | 18 | 7 | 6 |
| 14 | 11 | 2 | 5 |
| 15 | 19 | 6 | 6 |
| 16 | 14 | 3 | 5 |
| 17 | 12 | 5 | 2 |
| 18 | 12 | 4 | 0 |
| 19 | 7 | 1 | 1 |
| 20 | 14 | 4 | 6 |
| 21 | 5 | 5 | 1 |
| 22 | 3 | 2 | 2 |
| 23 | 4 | 3 | 2 |
| 24 | 5 | 1 | 0 |
| 25 | 2 | 2 | 2 |
| 26 | 3 | 1 | 3 |
| 27 | 3 | 0 | 1 |
| 28 | 0 | 1 | 0 |
| 29 | 2 | 1 | 0 |
| 30 | 2 | 1 | 1 |
| 31 | 0 | 0 | 1 |
| 32 | 0 | 1 | 0 |
| 35 | 1 | 0 | 0 |
| 37 | 1 | 1 | 0 |
| 43 | 1 | 0 | 0 |
| 46 | 1 | 1 | 0 |
| 48 | 0 | 1 | 1 |
| 51 | 0 | 0 | 1 |
