# Supplementary material for: LSD600: the first corpus of biomedical abstracts annotated with lifestyle–disease relations
Source: Database (Oxford). 2025 Jan 17;2025:baae129. doi: 10.1093/database/baae129 (PMC11756709; doi:10.1093/database/baae129)
Supplement: baae129_Supp [file baae129_supp.zip › suppl_data/SupplementaryTable5.docx]

Supplementary Table 5. Performance of the grid search on the development set, including hyperparameter values. The best model (highlighted in gray) is used to perform a run on the held-out test set.

| **MSL** | **LR** | **MaxEpochs** | **Precision** | **Recall** | **F-Score** |
| --- | --- | --- | --- | --- | --- |
| 180 | 5e-6 | 75 | **75.45** | **65.15** | **70.0** |
| 180 | 4e-6 | 75 | 70.25 | 66.15 | 68.0 |
| 128 | 5e-6 | 75 | 74.3 | 62.8 | 68.0 |
| 180 | 3e-6 | 75 | 72.15 | 60.45 | 65.8 |
| 180 | 5e-6 | 50 | 71.55 | 60.55 | 65.6 |
| 128 | 3e-6 | 75 | 72.35 | 59.5 | 65.3 |
| 128 | 4e-6 | 75 | 69.95 | 58.2 | 63.5 |
| 180 | 3e-6 | 50 | 64.85 | 60.5 | 62.5 |
| 128 | 2e-6 | 75 | 67.1 | 51.75 | 58.5 |
| 180 | 2e-6 | 75 | 58.9 | 53.9 | 56.3 |
| 128 | 1e-6 | 75 | 61.85 | 50.6 | 55.7 |
